# Accurate detection of influenza A virus by use of a novel cross-priming isothermal amplification-based point-of-care assay

**DOI:** 10.1128/spectrum.03074-23

**Published:** 2024-02-13

**Authors:** Jiankai Deng, Hongji Zhu, Shu An, Hao Huang, Ruizhi Wang, Yaoming Chen, Peisong Chen, Xuegao Yu

**Affiliations:** 1 Department of Laboratory Medicine, The First Affiliated Hospital, Sun Yat-sen University, Guangzhou, China; ^1^Foundation for Innovative New Diagnostics, Geneva, Switzerland

**Keywords:** influenza virus, point-of-care, cross-priming isothermal amplification, real-time RT-PCR, Xpert Xpress Flu/RSV assay

## Abstract

Influenza virus is known to cause respiratory tract infections of varying severity in individuals of all ages. The EasyNAT Rapid Flu assay is a newly developed *in vitro* diagnostic test that employs cross-priming isothermal amplification (CPA) to detect and differentiate influenza A and B viruses in human nasopharyngeal (NP) swabs. The aim of this study is to determine the performance characteristics of the EasyNAT Rapid Flu assay for rapid detection of influenza virus. The limit of detection (LOD) and cross-reactivity of the EasyNAT Rapid Flu assay were assessed. The clinical performance of the assay was evaluated using NP swab samples that were tested with real-time reverse-transcription polymerase chain reaction (RT-PCR) and Xpert Xpress Flu/RSV assay. The LOD for the detection of influenza A and B using the EasyNAT Rapid Flu assay was found to be 500 copies/mL. Furthermore, the assay exhibited no cross-reactivity with other common respiratory viruses tested. For the 114 NP swab samples tested for influenza A using both the EasyNAT Rapid Flu assay and real-time RT-PCR, the two assays demonstrated a high level of agreement (*κ* = 0.963, *P* < 0.001), with a positive percentage agreement (PPA) of 97.7% and a negative percentage agreement (NPA) of 98.6%. Similarly, for the 43 NP swab samples tested for influenza A and B using both the EasyNAT Rapid Flu assay and Xpert Xpress Flu/RSV assay, the two assays showed a high level of agreement (*κ* = 0.933, *P* < 0.001), with the overall rate of agreement (ORA) of 97.7% for influenza A and 100% for influenza B. The EasyNAT Rapid Flu assay demonstrates excellent performance in the detection of influenza A, highlighted by its strong agreement with RT-PCR-based assays.

**IMPORTANCE:**

The newly developed EasyNAT Rapid Flu assay is an innovative cross-priming isothermal amplification-based method designed for detecting influenza A and B viruses at point-of-care settings. This study aims to thoroughly assess the analytical and clinical performance of the assay, offering valuable insights into its potential advantages and limitations. The findings of this research hold significant implications for clinical practice.

## INTRODUCTION

Viral respiratory infections pose a significant health burden globally, particularly among vulnerable populations such as young children, pregnant women, older adults, and individuals with underlying health conditions (1, 2). Influenza virus is a major contributor to respiratory infections, especially in these high-risk groups (3–5). Among the influenza viruses, influenza A is the primary cause of epidemics, followed by influenza B. Timely and accurate viral diagnosis is crucial for patient management and infection control (6).

Diagnostic methods for viral infections have evolved over the years, progressing from conventional viral culture to more rapid techniques such as direct fluorescent antibody (DFA) and rapid antigen immunoassays, and most recently, highly sensitive nucleic acid amplification methods. Currently, laboratory-based real-time reverse-transcription polymerase chain reaction (RT-PCR) is considered the gold standard for influenza diagnosis. However, RT-PCR has limitations, including the need for specialized technical skills and a lengthy turnaround time (7). To address the need for more accessible testing to aid clinical decision-making, there has been a growing emphasis on point-of-care (POC) testing (8). POC nucleic acid amplification tests (NAATs) have rapidly emerged as a promising option. These tests have demonstrated the ability to reduce overall testing time, costs, isolation periods for hospitalized patients, and duration of hospitalization (9–11). Moreover, a positive POC test result for influenza prompts appropriate antiviral use and reduces unnecessary antibiotic prescriptions in patients with acute respiratory infections (12). Early initiation of antiviral treatment can shorten the duration of illness and lower the risk of developing severe complications (13).

Cross-priming isothermal amplification (CPA) is a highly effective and innovative method for nucleic acid amplification (14). It has been successfully utilized for detecting various pathogens, including infectious spleen and kidney necrosis virus, *Salmonella enterica* serovar Indiana, and *Staphylococcus aureus* (15–17). Similar to the EasyNAT MTC assay (18, 19) and the EasyNAT MP assays (20) developed by Ustar Biotechnologies in Hangzhou, China, the EasyNAT Rapid Flu assay is a newly developed CPA kit that can be performed on Ustar’s nucleic acid amplification and detection analyzer. This assay is designed for qualitative detection and identification of influenza A and B viruses in human nasopharyngeal (NP) swabs at POC. However, there are currently no published data on the performance characteristics of this assay.

On the other hand, the Xpert Xpress Flu/RSV assay, developed by Cepheid (Sunnyvale, CA, USA), is a real-time RT-PCR-based assay capable of detecting influenza A, influenza B, and respiratory syncytial virus (RSV). This assay has received Clinical Laboratory Improvement Amendments (CLIA) waivers for POC testing. With its fast turnaround time and user-friendly design, it enables molecular testing for samples requiring urgent testing, even during emergencies and non-office hours (21).

The objective of this study is to compare the performance characteristics of the EasyNAT Rapid Flu assay with those of the commercially available real-time RT-PCR and Xpert Flu/RSV Xpress assay for the detection of influenza virus in a clinical laboratory setting.

## MATERIALS AND METHODS

### The EasyNAT Rapid Flu assay used in this study

The EasyNAT Rapid Flu assay (Ustar Biotechnologies, Hangzhou, China) was performed on the Ustar’s nucleic acid amplification and detection analyzer (Ustar Biotechnologies, Hangzhou, China). The Ustar analyzer is a cartridge-based, automated real-time CPA instrument. The disposable cartridge, preloaded with reagents for the assay, can perform the entire nucleic acid analysis process directly on unprocessed clinical specimens (20). The target gene sequence of the influenza virus is amplified isothermally using specific primers within the cartridge. Fluorescence probes detect the presence of the influenza virus during the amplification process, and the analyzer automatically generates the test results. In the case of a positive test, a time threshold (Tt) value is provided, indicating the time taken for the fluorescence signal to reach a specified threshold.

In this study, the EasyNAT Rapid Flu assay was conducted following the manufacturer’s instructions. First, the Flu-RNA extraction solution (Ustar Biotechnologies, Hangzhou, China) was vortex-mixed for 30 seconds. After opening the cartridge cap, 1 mL of Flu-RNA extraction solution and 0.5 mL of the sample were transferred into the sample chamber. The cartridge cap was then covered, and the cartridge was gently shaken to ensure thorough mixing of the solution and the sample. Subsequently, the cartridge was inserted into the analyzer, which identified the pathogen-specific barcode on the cartridge and initiated the corresponding assay protocol. The hands-on time for the assay was less than 5 minutes, and the online detection time was approximately 38 minutes.

### Evaluation of the analytical performance of the EasyNAT Rapid Flu assay

The limit of detection (LOD) of the EasyNAT Rapid Flu assay for influenza A and B detection was determined using the BDS influenza A and BDS influenza B reference material kits (BDS Biotechnologies, Guangzhou, China). These kits contained non-replicative influenza A and influenza B viruses for absolute quantification. Four concentrations (5,000, 1,000, 500, and 100 copies/mL) were obtained by diluting the reference non-replicative viruses (15,000 copies/mL), and each concentration was tested with twenty replicates. The LOD was defined as the lowest dilution at which all replicates were detected as positive.

The cross-reactivity of the EasyNAT Rapid Flu assay was assessed against other common respiratory viruses, including rhinovirus (RhV), parainfluenza 1 virus, parainfluenza 2 virus, parainfluenza 3 virus, adenovirus, human metapneumovirus (HMPV), respiratory syncytial virus (RSV), and severe acute respiratory syndrome coronavirus 2 (SARS-CoV-2). Clinical isolates of RhV, parainfluenza 1/2/3 viruses, and adenovirus were donated by the Laboratory of Emerging Infectious Diseases and Division of Laboratory Medicine, Zhujiang Hospital (Guangzhou, China). Nucleic acid-positive clinical samples of HMPV, RSV, and SARS-CoV-2, confirmed by sequencing, were available from our laboratory. Each virus was tested twice, and the results that were not positive in both replicates were considered accurate.

### Evaluation of the clinical performance of the EasyNAT Rapid Flu assay

#### Clinical samples

**Fig 1:**
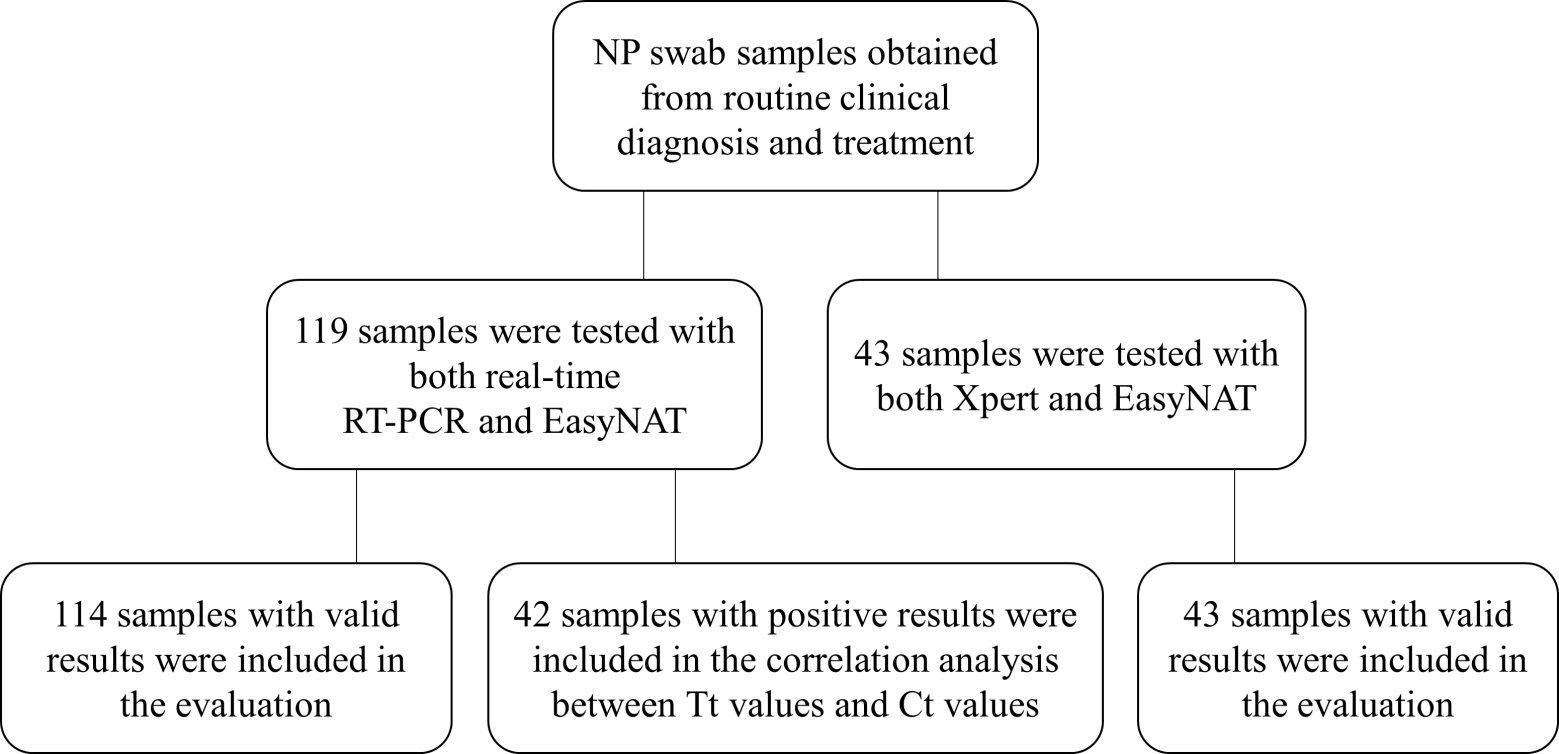
Diagram of the clinical sample processing scheme.

This study involved two distinct sets of retrospectively collected NP swab samples during the respiratory virus season from June 2022 to May 2023 (Fig. 1). The first set consisted of NP swab samples obtained from outpatients and emergency patients exhibiting influenza-like symptoms. As part of routine diagnostic procedures, these samples were collected in 3 mL universal transport medium (UTM) (DAAN GENE, Guangzhou, China) and subjected to influenza A testing using a commercially available real-time RT-PCR assay (DAAN GENE, Guangzhou, China). The second set comprised selected NP swabs in UTM that had undergone testing with the Xpert Flu/RSV Xpress assay (Cepheid, Sunnyvale, CA, USA) to ensure an adequate number of positive samples for influenza viral targets. After routine diagnostic assays were completed, the remaining UTM samples were stored at −80°C in aliquots for subsequent analysis using the EasyNAT Rapid Flu assay, which was conducted within 6 months.

This retrospective study utilized residual biological specimens obtained from routine clinical diagnosis and treatment. The study was approved by the Medical Ethics Committee of The First Affiliated Hospital, Sun Yat-sen University. Patients with a physician’s order for an influenza test were eligible to participate, and oral consent was obtained from the patients or their legal guardians.

#### Viral detection

All assays were performed following the manufacturer’s instructions. For the real-time RT-PCR assay, commercially available kits (DAAN GENE, Guangzhou, China) for influenza A detection were used. The process involved the extraction of nucleic acids, conversion of RNA to complementary DNA by reverse transcription, and detection by real-time PCR reaction on an ABI7500 system (Applied Biosystems, CA, USA). Two hundred microliters of the UTM sample was used for nucleic acid extraction using the Nucleic Acid Isolation or Purification Kit (DAAN GENE, Guangzhou, China). Nucleic acids were eluted in a volume of 50 µL, and 5.0 µL of the elution was used for each RT-PCR amplification. The cycle threshold (Ct) value, which is inversely related to viral load, was determined. A test was considered positive if the Ct value was ≤39.7.

The Xpert Xpress Flu/RSV assay was performed on the Cepheid GeneXpert Xpress system (Cepheid, Sunnyvale, CA, USA). Briefly, 300 µL of the UTM sample was transferred into the sample chamber of the cartridge using the provided disposable pipette. The cartridge lid was closed, and the cartridge was loaded into the instrument. The instrument identified the pathogen-specific barcode on the cartridge and initiated the assay. The hands-on time was less than 5 minutes, and the online detection time was approximately 33 minutes. In the case of a positive test, the Ct value, which is inversely correlated with viral load, was reported.

The EasyNAT Rapid Flu assay was performed on Ustar’s nucleic acid amplification and detection analyzer. If an invalid result was obtained with the initial test, the test was repeated once. If the invalid result recurred on the repeated test, it was recorded as a test failure.

### Analysis of the correlation between Tt values and Ct values

The correlation between Tt values and Ct values was evaluated using a dilution series of the BDS influenza A reference product (BDS Biotechnologies, Guangzhou, China), which contained non-replicative influenza A viruses (1,500,000 copies/mL) for absolute quantification. Five concentrations (1,000,000, 100,000, 10,000, 1,000, and 100 copies/mL) were tested with both the EasyNAT Rapid Flu assay and real-time RT-PCR. Additionally, the Tt values and Ct values were compared in clinical samples that tested positive for influenza A using both the EasyNAT Rapid Flu assay and real-time RT-PCR.

### Statistical analysis

The test results of the EasyNAT Rapid Flu assay were compared to those of real-time RT-PCR using positive percentage agreement (PPA), negative percentage agreement (NPA), and overall rate of agreement (ORA). The ORA between the EasyNAT Rapid Flu assay and the Xpert Flu/RSV Xpress assay was also calculated. Additionally, Cohen’s *kappa* (*κ*) was calculated to assess the overall agreement between the assays. Confidence intervals (CIs) were calculated using the Wilson score test. The correlation between Tt values and Ct values was analyzed using the Spearman correlation test. Statistical analyses were conducted using R version 3.6.1 within RStudio version 1.2.5. *P* values < 0.05 were considered statistically significant.

## RESULTS

### Analytical performance of the EasyNAT Rapid Flu assay

The analytical performance of the EasyNAT Rapid Flu assay was evaluated in terms of the LOD for influenza A and B detection, as well as cross-reactivity with other common respiratory viruses. The LOD evaluation demonstrated an LOD of 500 copies/mL for detection of both influenza A and B (Table 1). Regarding cross-reactivity, the EasyNAT Rapid Flu assay exhibited high specificity for the intended targets, showing no cross-reactivity with other common respiratory viruses (Table 2).

**TABLE 1:** Detection limit of the EasyNAT Rapid Flu assay

Target concentration, copies/mL	No. detected/No. of replicates	
	Influenza A	Influenza B
5,000	20/20	20/20
1,000	20/20	20/20
500	20/20	20/20
100	15/20	14/20

**TABLE 2:** Cross-reactivity of the EasyNAT Rapid Flu assay

Target	No. detected/No. of replicates
Rhinovirus	0/2
Parainfluenza 1 virus	0/2
Parainfluenza 2 virus	0/2
Parainfluenza 3 virus	0/2
Adenovirus	0/2
Human metapneumovirus	0/2
Respiratory syncytial virus	0/2
Severe acute respiratory syndrome coronavirus 2	0/2

### Clinical performance of the EasyNAT Rapid Flu assay

#### Comparison of the EasyNAT Rapid Flu assay with real-time RT-PCR

A total of 119 samples were tested for influenza A using both real-time RT-PCR and the EasyNAT Rapid Flu assay. Among them, ten samples initially yielded invalid results in the EasyNAT Rapid Flu assay, and after retesting, five samples produced valid results. Therefore, 114 samples with valid results were included in the analysis. The Ct values of these samples obtained from real-time RT-PCR ranged approximately from 21 to 39. Out of the total, 44 samples (38.6%) tested positive for influenza A, using at least one of the assays (Table 3). The EasyNAT Rapid Flu assay demonstrated a PPA of 97.7% (95% CI, 87.9%–99.6%) and an NPA of 98.6% (95% CI, 92.4%–99.8%) compared to real-time RT-PCR. The ORA between both assays was 98.2% (95% CI, 93.8%–99.5%). Two samples showed discordant test results (Table 4), with one having a high Ct value (36.5) and the other a high Tt value (19.8). The EasyNAT Rapid Flu assay achieved a detection yield of 100% for influenza A samples with Ct values below 35 and 90% for samples with Ct values greater than or equal to 35 (Table 5).

**TABLE 3:** Performance of the EasyNAT Rapid Flu assay compared with real-time RT-PCR^*a*^

EasyNAT	Real-time RT-PCR		PPA, % (95% CI)	NPA, % (95% CI)	ORA, % (95% CI)	*κ* (95% CI)	*P*
	Positive	Negative					
Influenza A			97.7 (87.9–99.6)	98.6 (92.4–99.8)	98.2 (93.8–99.5)	0.963 (0.911–1)	<0.001
Positive	42	1					
Negative	1	70					

^*a*^ RT-PCR, reverse-transcription polymerase chain reaction; CI, confidence interval; PPA, positive percentage agreement; NPA, negative percentage agreement; ORA, overall rate of agreement.

**TABLE 4:** Characteristics of patients with discordant test results between the EasyNAT Rapid Flu assay and real-time RT-PCR^*a*^

Test result	Tt value	Ct value	Duration of symptoms at the moment of sample collection, d	Antiviral therapy before sample collection	Age, y	Sex
EasyNAT+ / RT-PCR–	19.8	NA	10	No	13	M
EasyNAT– / RT-PCR+	NA	36.5	15	No	78	M

^*a*^ –, negative; +, positive; Tt, time threshold; Ct, cycle threshold; RT-PCR, reverse-transcription polymerase chain reaction; NA, not available; M, male.

**TABLE 5:** Positive rates of the EasyNAT Rapid Flu assay, stratified by real-time RT-PCR Ct values^*a*^

RT-PCR Ct value	Positive rate, %	95% CI, %
< 25	100 (4/4)	51–100
25–29.9	100 (19/19)	83.2–100
30–34.9	100 (10/10)	72.2–100
≥35	90 (9/10)	60–98.2

^*a*^ RT-PCR, reverse-transcription polymerase chain reaction; Ct, cycle threshold; CI, confidence interval.

#### Comparison of the EasyNAT Rapid Flu assay with the Xpert Xpress Flu/RSV assay

A total of 43 samples were tested using both the Xpert Xpress Flu/RSV assay and the EasyNAT Rapid Flu assay. The Ct values of these samples obtained from the Xpert Xpress Flu/RSV assay ranged approximately from 21 to 38. One sample initially yielded an invalid result in the EasyNAT Rapid Flu assay, but upon retesting, it produced a valid result. The ORA between the EasyNAT Rapid Flu assay and the Xpert Xpress Flu/RSV assay was 97.7% (95% CI, 87.9%–99.6%) for influenza A and 100% (95% CI, 91.8%–100%) for influenza B (Table 6). Only one sample showed a discordant test result, being positive for influenza A by the Xpert Xpress Flu/RSV assay but negative by the EasyNAT Rapid Flu assay. This sample was obtained from the patient 11 days after the onset of fever symptoms. Upon examination of the GeneXpert amplification plot of this discrepant sample, a very low level of fluorescence from the influenza A probe was observed at a Ct value of 34.9/36.9. No sample tested positive for influenza B in both assays.

**TABLE 6:** Performance of the EasyNAT Rapid Flu assay compared with the Xpert Xpress Flu/RSV assay^*a*^

EasyNAT	Xpert		ORA, % (95% CI)	*κ* (95% CI)	*P*
	Positive	Negative			
Influenza A			97.7 (87.9–99.6)	0.933 (0.802–1)	<0.001
Positive	9	0			
Negative	1	33			
Influenza B			100 (91.8–100)		
Positive	0	0			
Negative	0	43			

^*a*^ ORA, overall rate of agreement.

Furthermore, the EasyNAT Rapid Flu assay exhibited an overall detection time of less than 45 minutes, including hands-on time and online detection time, which is comparable to the approximately 40 minutes required by the Xpert Xpress Flu/RSV assay.

### Comparison between Tt values and Ct values

**Fig 2:**
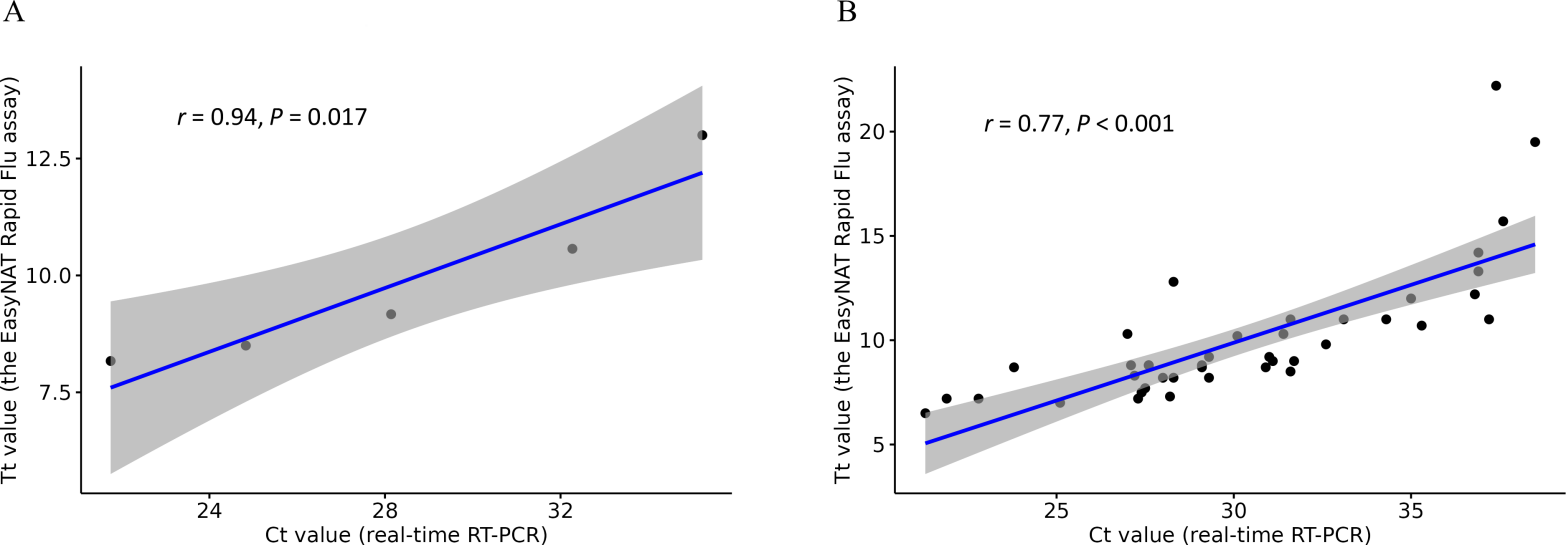
Scatterplots of the Tt values and Ct values for influenza A detection. (**A**) Comparison of the Tt values and Ct values in a dilution series of a reference sample. (**B**) Comparison of the Tt values and Ct values in 42 clinical samples.

Significant correlations were observed between Tt values (from the EasyNAT Rapid Flu assay) and Ct values (from real-time RT-PCR) for influenza A detection in both the dilution series of the influenza A reference sample (*r* = 0.94, *P* = 0.017; Fig. 2A) and 42 clinical samples (*r* = 0.77, *P* < 0.001; Fig. 2B).

## DISCUSSION

Influenza is a highly contagious respiratory tract infection that can cause varying degrees of symptoms in individuals of all ages. Early diagnosis of influenza virus infection is crucial for effective patient management and for reducing the burden on healthcare systems. Molecular POC assays, such as the EasyNAT Rapid Flu assay, offer rapid detection of influenza A and B viruses. This study aims to evaluate the performance of the EasyNAT Rapid Flu assay compared to that of real-time RT-PCR and the Xpert Xpress Flu/RSV assay.

The EasyNAT Rapid Flu assay utilizes isothermal nucleic acid amplification and can detect and differentiate between influenza A and B infections. It can be performed on Ustar’s nucleic acid amplification and detection analyzer, which has the capacity to process up to eight samples simultaneously. The assay exhibits reliable analytical performance, with a low LOD of 500 copies/mL and high specificity for influenza A and B detection. Additionally, the assay is fast, demonstrating comparable efficiency in sample processing and analysis to the Xpert Xpress Flu/RSV assay.

POC testing devices are necessary to improve human life with many aspects. According to the World Health Organization (WHO), an ideal POC device should satisfy the “ASSURED” criteria, which entails being affordable, sensitive, specific, user-friendly, rapid and robust, equipment-free, and deliverable to end-users (22). While the ASSURED criteria serve as a widely used principle for designing POC testing, it is challenging for all POC devices to fulfill each criterion. Previous studies have categorized the POC devices into different levels based on the setting (hospital or field) or budget (low or moderate), demonstrating that some ASSURED criteria may not be essential in certain specific cases (23). POC diagnostic devices can be tailored differently depending on the intended use. The EasyNAT Rapid Flu assay, designed for POC testing for influenza A and B viruses, exhibits sensitivity, specificity, ease-of-use, and rapidity. However, it does not fully meet the criteria of an ideal POC test, as the assay can only be performed on Ustar’s nucleic acid amplification and detection analyzer. Furthermore, although this assay simplifies traditionally complex laboratory processes, it still requires a few pipetting steps that are best executed by trained personnel. Nevertheless, the EasyNAT Rapid Flu assay shows promise as a complementary tool to traditional laboratory-based RT-PCR assays for diagnosing influenza in a hospital setting.

In this study, the EasyNAT Rapid Flu assay demonstrated a high PPA of 97.7% and ORA of 98.2% compared to real-time RT-PCR for influenza A detection in a clinical laboratory setting. Additionally, there was a high agreement (*κ* = 0.933, *P* < 0.001) between the EasyNAT Rapid Flu assay and the Xpert Xpress Flu/RSV assay in detecting both influenza A and B. Only three samples exhibited discordant test results for influenza A detection, all of which displayed high Ct values (real-time RT-PCR 36.5, Xpert Xpress Flu/RSV 34.9/36.9) or a high Tt value (19.8). This indicates that the influenza A targets may potentially be present at extremely low levels in these samples. These particular samples were obtained from patients experiencing a prolonged duration of symptoms (≥10 days), and it is reported that influenza A viral RNA concentration diminishes non-linearly over time since symptom onset (24). Hence, the discordant test results likely reflect the detection variability due to the low concentration of the target near its LOD, rather than a difference in assay performance.

Instead of using sensitivity and specificity to describe concordance between both assays, we employed PPA and NPA to characterize the performance of the EasyNAT Rapid Flu assay compared to that of real-time RT-PCR. While real-time RT-PCR is widely accepted as the gold standard for virus detection, no assay achieves 100% accuracy. Percentage agreement is recommended by the US Food and Drug Administration (FDA) when comparing a new test to an imperfect reference test (25).

There are several limitations to this study. First, the assays were performed at different time points, which may have affected the viral load of the samples and led to differences in test results. However, all NP swabs in UTM were stored at −80°C until testing, ensuring long-term sample storage without significant effects on sample quality. Second, the number of included samples was relatively low. Nonetheless, the high concordance between the assays across a representative range of Ct values provides confidence in the reliability of the results. Lastly, due to the absence of positive samples for influenza B during the study, the performance of the EasyNAT Rapid Flu assay for detecting influenza B could not be assessed.

The CT value in RT-PCR serves as a surrogate marker of viral load, with lower values indicating higher viral concentrations. Previous studies have shown that higher viral loads of SARS-CoV-2 are associated with severer COVID-19 symptoms (26, 27) and increased mortality (28, 29). In this study, a significant correlation was observed between Tt values and Ct values. Further research is needed to determine if assessing influenza viral load through Tt values in the EasyNAT Rapid Flu assay can be a clinically valuable tool in evaluating the severity of influenza presentation.

In conclusion, this study provides the first clinical evaluation of the EasyNAT Rapid Flu assay for influenza virus detection, comparing it to real-time RT-PCR and the Xpert Xpress Flu/RSV assay. The assay demonstrates excellent agreement with these RT-PCR-based assays, offering a fast solution. With its demonstrated good diagnostic performance, the EasyNAT Rapid Flu assay shows promising potential in aiding accurate diagnosis of influenza.
